# HCV Genome-Wide Genetic Analyses in Context of Disease Progression and Hepatocellular Carcinoma

**DOI:** 10.1371/journal.pone.0103748

**Published:** 2014-07-31

**Authors:** Maureen J. Donlin, Elena Lomonosova, Alexi Kiss, Xiaohong Cheng, Feng Cao, Teresa M. Curto, Adrian Di Bisceglie, John E. Tavis

**Affiliations:** 1 Department of Molecular Microbiology and Immunology, Saint Louis University School of Medicine, Saint Louis, Missouri, United States of America; 2 Edward A. Doisy Department of Biochemistry and Molecular Biology, Saint Louis University School of Medicine, Saint Louis, Missouri, United States of America; 3 Saint Louis Liver Center, Saint Louis University School of Medicine, Saint Louis, Missouri, United States of America; 4 Department of Internal Medicine, Saint Louis University School of Medicine, Saint Louis, Missouri, United States of America; 5 New England Research Institutes, Inc., Watertown, Massachusetts, United States of America; University of Montreal Hospital Research Center (CRCHUM), Canada

## Abstract

Hepatitis C virus (HCV) is a major cause of hepatitis and hepatocellular carcinoma (HCC) world-wide. Most HCV patients have relatively stable disease, but approximately 25% have progressive disease that often terminates in liver failure or HCC. HCV is highly variable genetically, with seven genotypes and multiple subtypes per genotype. This variation affects HCV’s sensitivity to antiviral therapy and has been implicated to contribute to differences in disease. We sequenced the complete viral coding capacity for 107 HCV genotype 1 isolates to determine whether genetic variation between independent HCV isolates is associated with the rate of disease progression or development of HCC. Consensus sequences were determined by sequencing RT-PCR products from serum or plasma. Positions of amino acid conservation, amino acid diversity patterns, selection pressures, and genome-wide patterns of amino acid covariance were assessed in context of the clinical phenotypes. A few positions were found where the amino acid distributions or degree of positive selection differed between in the HCC and cirrhotic sequences. All other assessments of viral genetic variation and HCC failed to yield significant associations. Sequences from patients with slow disease progression were under a greater degree of positive selection than sequences from rapid progressors, but all other analyses comparing HCV from rapid and slow disease progressors were statistically insignificant. The failure to observe distinct sequence differences associated with disease progression or HCC employing methods that previously revealed strong associations with the outcome of interferon α-based therapy implies that variable ability of HCV to modulate interferon responses is not a dominant cause for differential pathology among HCV patients. This lack of significant associations also implies that host and/or environmental factors are the major causes of differential disease presentation in HCV patients.

## Introduction

Hepatitis C virus (HCV) is a Hepacivirus that infects hepatocytes and some lymphocytes [1], [2]. It chronically infects about 120–170 million people world-wide, resulting in about 350,000 deaths annually [3], [4]. Disease caused by HCV ranges from asymptomatic infection to severe hepatitis, with most people having some degree of ongoing liver damage [1], [5]. Roughly 25% of chronically infected individuals have progressive disease, where liver pathology proceeds from hepatitis of gradually worsening severity, to hepatic fibrosis, cirrhosis, and often to fatal liver failure or hepatocellular carcinoma (HCC). The rate of progression along this spectrum varies from a few years in exceptionally rapid progressors to many decades in slow progressors, with relatively slow disease progression being the norm. HCV-induced liver disease is primarily caused by hepatic inflammation and anti-HCV immune responses [6]–[8]. Direct cytopathic effects from viral replication may contribute to disease, but they are believed to be secondary to immune-mediated damage.

**Figure 1:**
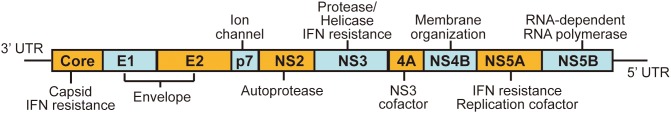
HCV genome. The HCV genome contains 5′ and 3′ untranslated regions and a single, long open reading frame that encodes 10 proteins. The mature viral proteins encoded within the open reading frame and their major functions are indicated. Reprinted from [25] under the creative commons license.

HCV’s ∼9,600 nucleotide positive-polarity RNA genome encodes a polyprotein of ∼3100 amino acids that is cleaved into 10 mature proteins (Fig. 1). The genome is surrounded by a capsid composed of the viral core protein, and the capsid is enclosed by a lipid envelope containing the viral glycoproteins E1 and E2. The non-structural proteins (P7-NS5B) replicate the viral RNA, and virions are secreted from the cell non-cytolytically [9], [10]. The HCV genome is highly variable, with seven genotypes that are less than 72% identical at the nucleotide level [11]. Within the genotypes, subtypes with nucleotide identities of 75–86% may occur. Individual isolates of a given subtype are typically ∼92–96% identical, and as HCV replicates as quasispecies, multiple variants differing by up to a few percent exist within individual patients. The viral 5′ untranslated region, the core gene, and the extreme 3′ end of the genome are relatively well conserved, and two hypervariable regions within the envelope proteins, the 3′ end of the NS5A gene, and parts of the 3′ untranslated region are very poorly conserved.

Until recently the standard treatment for chronic HCV infection was pegylated interferon α (IFNα) plus ribavirin for 24 to 48 weeks, which resulted in clearance of the virus [sustained viral response (SVR)] in 50–60% of genotype 1 patients [12], [13]. In 2011, two inhibitors of the HCV NS3 protease, telaprevir and boceprevir, were approved for use in conjunction with interferon α in HCV genotype 1 patients that improved SVR rates to ∼75% [14], [15]. A third inhibitor of the NS3 protease (simeprevir) and a nucleoside analog that targets the NS5B RNA polymerase (sofosbuvir) were approved in 2013 [16]–[18], increasing efficacy of the triple-therapy combinations. However, stimulation of the interferon response remains key to efficacy of the existing triple therapies, and HCV treatment will remain dependent on interferon α until sets of direct-acting drugs with sufficient efficacy to eradicate the virus by themselves is approved, as is expected to happen [19].

HCV’s genetic variation has a major impact on success of both interferon α-based therapy and direct inhibitor-based treatments. Telaprevir and boceprevir are approved exclusively for patients infected with HCV genotype 1 [20], whereas simeprevir is approved for use against both genotype 1 and 4 infections [16]. Most experimental direct-acting agents are also genotype-specific [21], [22]. Interferon plus ribavirin therapy clears genotype 1 infections much less well than genotype 2 and 3 infections (∼50% compared to >80%, respectively) [23], [24]. We previously found that high genetic variation in the consensus sequences of the HCV core, NS3, and NS5A genes was tightly correlated with failure of interferon α plus ribavirin therapy [25]–[27]. Importantly, all three of these genes can counteract the type 1 interferon response [28]. We interpreted this association to indicate that high viral variability impairs the ability of HCV’s interferon-suppressive proteins to counteract the heightened type 1 interferon responses induced by therapy. We and others also found that ∼10% of HCV’s ∼3000 amino acid positions covary with at least one other position, and that these covariances link together into a genome-wide network of covarying positions [27], [29]. These networks are different among HCV sequences from responders and non-responders to interferon plus ribavirin therapy [27], [30], [31], implying a coordinated role for sequence variation throughout the viral genome in antagonizing interferon responses.

The impact of HCV genetic variation on viral pathology is less clear. It is well accepted that genotype 3 causes steatosis more frequently than the other HCV genotypes [32], [33]. Furthermore, HCV infection can elevate levels of the pro-inflammatory cytokine IL8 [34] through activation of the IL8 promoter by core, NS4B, and/or NS5A [35]–[37], and there is a direct correlation between core sequence variation, ALT levels, and IL8 promoter activation [38]. Other associations between HCV genetic variation and pathology are less well accepted. Some studies found little evidence for virulence differences between the major HCV genotypes [39], [40], but most studies found differences, such as genotype 1 being more virulent than genotype 2 [41], [42]. Most studies have found genotype 1b to be more virulent and more highly associated with HCC than other genotypes [43]–[47]. However, these associations have not been apparent in other studies [48], [49], and some of the higher virulence of 1b has been suggested to be due to accidental selection bias in the patient populations [50].

Stronger evidence exists for a role of HCV genetic variation on HCC development. Most genetic analyses of HCV in the context of HCC have focused on the HCV core and NS5A genes. The HCV core protein has been reported to promote cellular transformation in tissue culture [51] and in some animal models [52], [53], and several studies have found an association between variations in the core coding sequence and the likelihood of developing HCC [54]–[58]. Akuta et. al identified two core amino acid positions (70 and 91) where non-wild type residues were significantly associated with HCC in genotype 1b patients [55]–[57]. Furthermore, Fishman et. al. examined core nucleotide positions and their putative effect on known RNA structures in subtype 1b and identified several positions where substitutions were associated with increased risk of HCC [54]. Inhibition of PKR activity by the NS5A PKR binding site has been shown to be needed for cellular transformation and tumorigenicity in nude mice [59], but both low [60], [61] and high [62] diversity of the PKR binding site have been associated with HCC. Studies in which HCV variation has been described at just the subtype level found that 1b is associated with a higher risk of HCC than 1a [46], [47], [63]. Three studies examined full-length HCV genomes at the sequence level in the context of HCC [64], [65] [66], and each study identified a small number of amino acid positions where genetic variation was significantly associated with HCC.

We hypothesized that HCV genetic variation may be associated with differential virulence in HCV, specifically with the rate of advancement of liver disease and/or development of HCC. This hypothesis was based on our identification of clear HCV genetic patterns that were associated with outcome of interferon-based antiviral therapy [25]–[27]. The null hypothesis was that environmental and/or host-specific factors were dominant in determining the differential disease outcomes. Two independent patient sets were employed to assess this hypothesis. The first set was used to evaluate association of HCV genetic variation with development of HCC. These patients were identified through the Liver Cancer Research Network (LCRN; [67]). The second set was used to assess the role of HCV genetic diversity in the rate of disease progression. These patients were derived from the untreated observational control arm of the HALT-C clinical trial, which was a multi-center, randomized controlled study designed to determine if long-term interferon α treatment would ameliorate HCV’s pathology [68]. Our strategy was to determine the consensus sequence for the full HCV coding region by direct sequencing of nested reverse-transcription-PCR products, and then to compare HCV genetic patterns in HCC vs. non-HCC patients for the cancer cohort or the slow vs. rapid progressors from the HALT-C cohort.

## Methods

### Ethics Statement

This study was approved by the Saint Louis University Biomedical Institutional Review Board (HCC cohort, IRB#1570; HALT-C cohort IRB#14138). All participants provided written informed consent to participate in the parent HALT-C and LCRN studies; this informed consent included granting permission for use of de-identified samples for study-approved ancillary studies such as this. This informed consent procedure was approved by the IRBs for the parental study, and each patient’s informed was documented and filed by the parental studies.

### Sequencing the HCV open reading frame

HCV RNAs were isolated from patient serum and cDNAs were synthesized as previously described [69], [70]. For the HCC samples and cirrhotic controls, cDNAs were sequenced with the nested reverse transcriptase-PCR and direct sequencing methods we previously employed for Virahep-C samples [26], [69]. cDNAs from the HALT-C samples were sent to the Broad Institute for nested reverse transcriptase-PCR and direct sequencing of the overlapping amplicons by the chain-termination method as described [70]. Approximately 50% of the viral sequence data were obtained by this approach. The remaining data for the HALT-C sequences were obtained employing our higher-sensitivity nested reverse transcriptase-PCR and direct sequencing methods [26], [69]. The extreme 3′ end of the HCV open reading frame could not be obtained for all patients. Consequently, the sequences were truncated at aa 8991 for the cancer cohort and at aa 8994 for the HALT-C patients to ensure equal coverage of all genomic regions in the analyses. This eliminated the 14 C-terminal codons for the cancer cohort and the 13 C-terminal codons for the HALT-C sequences. Genbank numbers for HCV sequences from the HCC cohort are: KC439481–KC439502 (HCC) and KC439503–KC439527 (cirrhotic controls). Genbank numbers for HCV sequences from the HALT-C patients are: JX463525–JX463554 (time point 1, rapid progressors); JX463555–JX463584 (time point 1, slow progressors); JX463585–JX463612 (time point 2, rapid progressors), and JX463613–JX463641 (time point 2, slow progressors). The list of sequence IDs, accession numbers and experimental groups for both patient cohorts are in Table S1.

### Clonal sequencing in the E2 gene

Twelve clones encompassing the amino-terminal region of the E2 glycoprotein (aa 384–476 in the HCV polyprotein) that included the hypervariable region 1 (aa 384–410) from each of six HCC and six cirrhotic control patients were cloned for quasispecies analyses. HCV RNAs were isolated and cDNA was synthesized as was done for the direct sequencing. HCV sequences were amplified by nested PCR from the cDNAs under high-fidelity conditions employing the Hotstart HiFidelity Polymerase kit (Qiagen). The PCR products were cloned and independent clones were randomly selected for sequencing.

### Sequence analyses

All analyses except dinucleotide frequency analyses and codon selection biases were conducted at the amino acid level. Consensus population-wide reference sequences were derived from 107 full-length genotype 1b or 103 genotype 1a ORFs downloaded from Genbank in January, 2012. Sequence alignments were done with Muscle [71]. Positions that varied relative to the genotype 1a or 1b reference consensus sequences were identified with the EMBOSS program Infoalign 4501 [72]. Mean genetic distance was calculated using the p-distance algorithm in the MEGA v. 5 DNA analysis package [73]. The codon selection analysis based on the ratio of dN/dS substitutions was done using the single likelihood ancestor counting (SLAC) method with the HKY85 substitution mode and a significant level of p<0.05 [74]. The predicted frequency of specific dinucleotide pairs within an ORF was calculated by multiplying the frequency in the ORF of both bases in the pair by the length of the ORF using customized PERL scripts. The observed base and dinucleotide compositions were counted directly using customized PERL scripts.

### Amino acid covariance analyses

All possible amino acid covariances within the HCV open reading frame were determined employing the observed-minus expected-squared algorithm with a 1% false discovery rate as we have previously described [27], [75]. Networks in the covariance data were graphed employing Cytoscape [76]. Network metrics were calculated employing the Network Analyzer plug-in for Cytoscape [77].

### Statistical analyses

Positions of skewed amino acid variance between the groups of sequences were identified by comparing positions of variance in each group with a Mann-Whitney ranked sums test. Differences in the average protein distances, the average number of variations/sequence and dinucleotide frequencies were compared with a t-test. Statistical analyses were carried out using SPSS v19 (IBM Corporation, Armonk, NY). Baseline variables in the rapid and slow progressor groups were compared using the chi-square test, the t-test, or the Wilcoxon rank-sum test using SAS v.9.3 (SAS Institute, Cary, NC).

## Results

### Patient selection and sequencing to evaluate association of HCV genetic variation with HCC

Fifty patients were identified through the Liver Cancer Research Network (LCRN) and Dr. Di Bisceglie’s practice at Saint Louis University for the cancer cohort. All patients were infected with HCV subtype 1b and had a clinical diagnosis of cirrhosis at or prior to sample collection. Exclusion criteria included co-infection with HBV or HIV, evidence of alcohol abuse, and evidence of other liver diseases including non-alcoholic fatty liver disease or hemochromatosis. “*HCC patients*” had a definite or presumed HCC diagnosis at sample collection. Definite HCC was biopsy-proven HCC or the presence of a new defect within the liver noted on imaging studies with a serum alpha-fetoprotein (AFP) level of >1,000 ng/ml. Presumed HCC was three separate imaging techniques suggestive of HCC, a new hepatic defect followed by massive hepatic involvement and death, or a new hepatic defect with increasing size or increasing serum AFP. “*Cirrhotic controls*” were cirrhotic (confirmed by liver biopsy, with Metavir score ≥4) but had no clinical evidence of HCC at the time of sample collection. HCC was excluded in the controls by routine ultrasound surveillance every 6 to 12 months according to the AASLD practice guideline on management of HCC. The HCC and cirrhotic control patient groups were matched by age and sex. The annual incidence rate of HCC in cirrhotic HCV-infected patients is 1 to 4% [78]. Therefore, our power calculations assumed that two of the 25 cirrhotic controls (8%) that were cancer-free at sample collection would develop HCC within a few years. Using 25 controls yielded >80% power at α = 0.05 to detect genetic differences similar to what we had observed with the Virahep-C samples between the HCC and cirrhotic groups, even with this high degree of contamination of the controls.

Consensus sequences for the full HCV coding region were obtained from serum-derived RNA employing the nested reverse transcriptase-PCR and direct sequencing methods we previously employed [26], [69]. We were unable to sequence the full coding region from three HCC patients so these sequences were excluded. The HCC and cirrhotic control groups remained statistically indistinguishable for age and sex following exclusion of these three patients (Table 1).

**Table 1:** Age and gender of patients from whom the HCC and cirrhotic samples were derived.

	Cirrhotic control	HCC	P value^1^
Number of patients	25	22	–
Gender (M/F)	19/6	16/6	ns
Age (mean ± SD)	57.4±9.6	61.7±8.2	ns
Age (range)	49–76	44–82	ns

1 ns, non-significant.

### HCV positional sequence differences associated with HCC

To identify amino acid positions in the HCV sequence that differed consistently between the HCC and cirrhotic control sequences, we aligned the sequences and examined amino acid distributions at all 2997 positions. The amino acid distributions at 25 aa positions were significantly different between the HCC and cirrhotic control samples, with p-values ranging from 0.001 to 0.046 (Table 2). As a control to determine the frequency of chance associations in this analysis, the 47 sequences were randomly re-sorted into five sets of 25 and 22 sequences and positions where the amino acid distribution differed significantly between these pairs of biologically irrelevant groups were identified. We observed a mean of 15.2 (10–22) positions that differed with a mean p-value of 0.025 (0.001 to 0.049) in these control comparisons. The larger number of significantly different positions in the HCC versus cirrhotic case compared to the scrambled control sequence sets suggests some of the 25 positions of skewed variance between HCC and cirrhotic controls may be associated with a biological difference between the two groups. Four of the positions that were significantly associated with HCC occurred in the very small (63 residue) p7 gene.

**Table 2:** Positions where the distribution of amino acids differ in HCC and cirrhotic control sequences.

Position	Protein	P value
75	Core	0.038
274	E1	0.015
330	E1	0.033
438	E2	0.020
476	E2	0.025
496	E2	0.028
524	E2	0.026
538	E2	0.046
699	E2	0.013
741	p7	0.044
759	p7	0.019
760	p7	0.018
767	p7	0.028
938	NS2	0.046
1087	NS3	0.016
1323	NS3	0.018
1329	NS3	0.008
1536	NS3	0.012
1694	NS4A	0.038
2016	NS5A	0.029
2278	NS5A	0.031
2356	NS5A	0.046
2385	NS5A	0.028
2543	NS5B	0.001
2650	NS5B	0.014

### HCV consensus sequence diversity differences are not associated with HCC

To determine if there were diversity differences between the HCC and cirrhotic control sequences, pairwise genetic distances between all samples within the HCC and cirrhotic controls groups were calculated, and then the average pairwise distances were compared between the two groups. The mean pairwise differences for the HCC and cirrhotic control groups (0.081 vs. 0.080, respectively) were not significantly different. A more sensitive method to measure genetic diversity is to quantify the number of variations relative to a population-wide consensus reference sequence for each sample. Therefore, each sample was aligned to a subtype 1b population-wide reference sequence, and the number and identity of variations were recorded for each sample as we have done before [25], [26], [79]. No significant differences were found between the HCC and cirrhotic controls for either total number of variations in the two groups or the number of variations that were unique to either the HCC or cirrhotic groups. This held true when the entire polyprotein was evaluated as a single unit and when the viral genes were considered individually.

### HCV quasispecies patterns in the E2 HVR region are not strongly associated with HCC

To determine if there were genetic differences between the HCC and cirrhotic groups at the quasispecies level, we sequenced 12 independent clones covering the 27 amino acid-long E2 hypervariable region (HVR) plus 66 amino acids downstream of the HVR from each of six randomly-selected patients in both the HCC and cirrhotic groups. The number of amino acid differences relative to a genotype 1b population reference per patient was not significantly different between sequences from the HCC and cirrhotic samples. Amino acid pairwise distances were determined within the set of 12 sequences for each patient as a measure of the quasispecies diversity. The mean pairwise protein genetic differences were slightly higher in the E2 region (0.066 vs. 0.036) and the HVR region (0.278 vs. 0.148) for the HCC samples compared to the cirrhotics. Sequence complexity within the 12 sequences per patient was also assessed. The HCC patients had an average of 9.2 unique E2 sequences per patient compared to 7.2 for the cirrhotic controls, and the HCC samples had an average of 7.5 unique HVR sequences per patient compared to 6.5 for the cirrhotics. Similar results were obtained when the data were analyzed at the nucleotide level. Thus, the HCV sequences in the HCC patients appeared to be slightly more diverse and complex than in the cirrhotic controls, but these differences were not statistically significant. There was no evidence of positive selection in these sequences. Overall, no prominent differences in the quasispecies spectra in the HCC and control patients were detected.

### Selective pressures associated with HCC

We examined the HCC and cirrhotic control sequences for differences in selective pressure at all 2997 codons using the SLAC method with the HKY85 substitution mode in order to identify the codons under positive or negative selection [74]. 825 of the 2997 codons were under negative selection and 12 codons were under positive selection in the HCC sequences, while 900 codons were under negative selection and 13 codons under positive selection in the cirrhotic controls (Table 3). Only three of the positively-selected codons were shared between the two groups.

**Table 3:** Codons under positive selection in the HCC and cirrhotic control sequences.

Codon	Gene	Group	dN-dS	P value
**75**	**Core**	**Cirrhotic**	**2.341**	**0.039**
384	E2	Cirrhotic	6.042	0.008
387	E2	Cirrhotic	3.455	0.039
397	E2	Cirrhotic	5.512	0.011
401	E2	Cirrhotic	5.514	0.011
**476**	**E2**	**Cirrhotic**	**3.650**	**0.012**
478	E2	Cirrhotic	4.741	0.021
479	E2	Cirrhotic	3.470	0.030
522	E2	Cirrhotic	4.359	0.005
**1384**	**NS3**	**Cirrhotic**	**2.630**	**0.026**
**2278**	**NS5A**	**Cirrhotic**	**2.632**	**0.026**
2968	NS5B	Cirrhotic	4.578	0.002
2983	NS5B	Cirrhotic	2.804	0.015
387	E2	HCC	3.283	0.026
401	E2	HCC	4.197	0.004
**407**	**E2**	**HCC**	**3.032**	**0.017**
434	E2	HCC	3.795	0.013
461	E2	HCC	3.025	0.017
522	E2	HCC	2.811	0.036
**837**	**NS2**	**HCC**	**3.289**	**0.044**
**962**	**NS2**	**HCC**	**3.617**	**0.020**
**1098**	**NS3**	**HCC**	**3.010**	**0.018**
2632	NS5B	HCC	3.983	0.009
2983	NS5B	HCC	4.187	0.001

**Bold** indicates positions unique to the HCC or cirrhotic sequence alignments compared to alignments of randomly selected HCV control sequences.

To help evaluate whether these selective differences may be associated with disease state or may simply represent selective pressures on the HCV population as a whole, we randomly sampled six sets of 22 or 25 HCV 1b coding sequences of the same length (2997 codons) from Genbank and examined them for codon selection differences. Six of the 25 positions under positive selection in the HCC or cirrhotic control sequences were not under positive selection in any of the six randomly selected sequence sets (bold in Table 3). This indicates that the positive selection pressures on most of the sites we identified were probably unrelated to the patient’s disease state, but that selection at the six codons unique to the HCC or cirrhotic patients may reflect evolutionary pressures associated with these advanced disease states.

### UU and UA dinucleotide frequency differences are not associated with HCC

RNase L is an endoribonuclease that cleaves RNA at single-stranded UA and UU dinucleotides [80]. RNAse L contributes to the innate immune responses against many viruses. We and others have shown that RNase L exerts evolutionary pressure on HCV genomes, as evidence by a reduced frequency of UU and UA dinucleotides than would be expected by chance [26], [81]. We extended these analyses by determining the ratio of observed/predicted dinucleotide frequencies for every possible dinucleotide pair for each of the HCV sequences from the HCC and cirrhotic patients. All of the samples showed the predicted reduced frequency of UA and UU dinucleotide pairs, with an average observed/expected ratio of 0.81 and 0.94 respectively. However, there were no significant differences in the frequency of any dinucleotide pair between HCC and the cirrhotic controls.

### Amino acid covariance patterns associated with HCC

**Figure 2:**
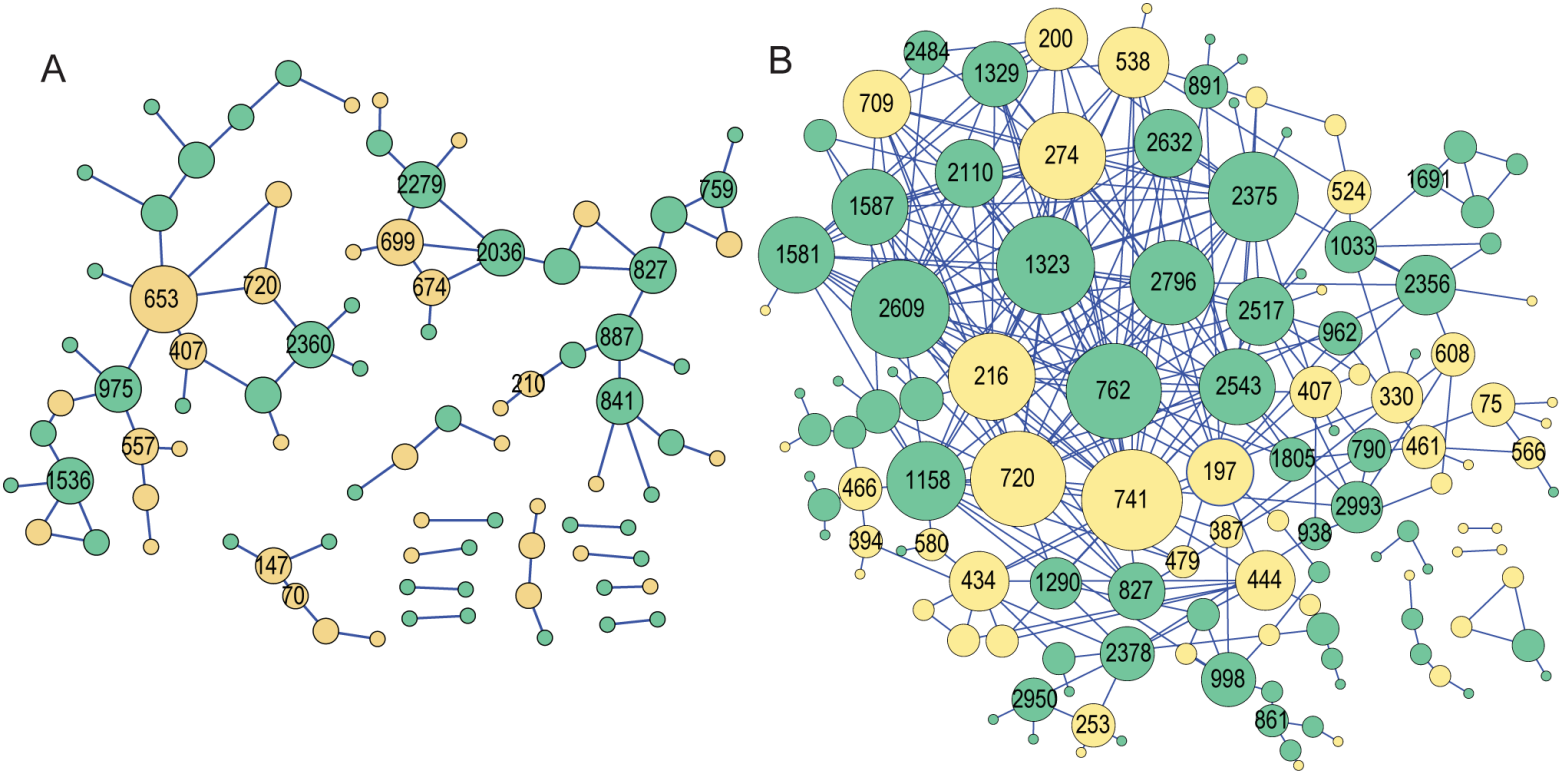
Amino acid covariance networks for the HCC and cirrhotic sequences. Amino acid covariances within alignments of the HCV cirrhotic (left) and HCC (right) sequences were graphed with the covarying positions (nodes) represented as circles and the covariances between the positions (edges) as lines. The size of the nodes is proportional to the number of edges that they contact. Yellow nodes are within structural proteins and green nodes are in non-structural proteins. The amino acid residue position numbered relative to the HCV polyprotein is indicated in the larger nodes.

Next, we asked whether differences in genome-wide amino acid covariance networks distinguished the HCC and cirrhotic control sequences. This analysis was based on our previous detection of prominent differences in the networks from responders and non-responders to interferon-based therapy [27], [31]. Amino acid covariance networks were generated for the HCC and cirrhotic controls as previously described [75]. As has been observed for other HCV sequence sets [27], [29], [31], [75], covariance networks containing residue positions from all 10 proteins that had a hub-and-spoke topology were observed. However, the HCC network had many fewer nodes and was much less tightly connected than the cirrhotic network (Table 4). The less-connected nature of the HCC network was obvious visually, as it formed two major and many smaller networks instead of a single large network as was formed by the cirrhotic sequences (Fig. 2).

**Table 4:** Basic amino acid covariance network parameters for the HCC and cirrhotic sequences.

Network	Number of sequences	Number of nodes	Number of edges	Average number of neighbors	Network density
Experimental sequences					
HCC	22	86	80	1.9	0.022
Cirrhotic	25	128	296	4.6	0.036
Randomly sampled sets of 22 cirrhotic sequences					
Set 1	22	123	320	5.2	0.043
Set 2	22	120	255	4.2	0.036
Set 3	22	118	309	5.2	0.045
Set 4	22	121	327	5.4	0.045
Set 5	22	118	215	3.6	0.03
Set 6	22	112	160	2.8	0.03
External HCC sequence sets					
Pooled set 1	22	116	505	8.7	0.76
Pooled set 2	22	138	663	9.6	0.07
Takahashi et al.^1^	15	88	441	10	0.12
Nagayama et al.^2^	13	99	195	3.9	0.04

1 Sequences obtained from [65].

2 Sequences obtained from [64].

We previously found that detection of covariances was sensitive to the number of sequences employed, with 22–25 sequences being on the lower end of the useful range [75]. Therefore we asked if this network integrity difference was due to the fewer number of sequences in the HCC set by randomly selecting six sets of 22 cirrhotic sequences and generating analogous amino acid covariance networks. All of the networks contained very similar numbers of nodes and covarying pairs as were found in the network with 25 cirrhotic sequences, suggesting that the network is robust to the loss of a few sequences (Table 4). To test the possibility that the network connectivity differences were due to a random sampling of HCC sequences, we generated analogous covariance networks from two other sets of full-length HCV sequences from HCC-positive patients [64], [65] (15 or 13 sequences). We also combined these two sequence sets and generated two networks from randomly selected sets of 22 sequences from the combined set of 28 sequences; Table 4). In all 4 of these HCV covariance networks derived from HCC patients outside of our patient cohort, the covariances formed a single, highly connected network with network parameters similar to the cirrhotic network and to the previously published HCV networks [27], [75]. This suggests that the fragmented covariance network observed with our HCC sequences is unlikely to be a general feature associated with HCC patients.

### Positional differences in the HCV core gene associated with HCC

Genetic variations at 11 nucleotide [54] and two amino acid [55]–[57] positions in the core gene have been associated with HCC. Therefore, we evaluated these genetic signatures in our sequences. The HCC and the cirrhotic control sequences both predominantly carried the control-type rather than the HCC-type sequences at 8 of the 11 sites that were associated with HCC by Fishman et al. [54] (Table 5). Furthermore, the distribution of HCC- and control-type sequences at all of these positions was quite similar in our HCC and cirrhotic sequence sets. Sequence data for four of these positions are available for an independent set of cirrhotic patients [62]. The sequence patterns in this independent cirrhotic cohort were nearly identical to the patterns we observed at all 4 positions (Table 5).

**Table 5:** Positions in core associated with HCC.

	Residue identity		Control-type/HCC-type residues (number of sequences)		
Residue number^1^	Control-type	HCC-type	Cirrhotic sequences	HCC sequences	External cirrhotic sequences^2^
**Nucleotide positions** 3					
36	A	G/C	25/0	21/1	22/5
78	U	C	1/24	2/20	1/25
209	G	A	7/18	5/17	6/19
271	U/C	A	3/22	5/17	6/19
309	U	C/A	21/4	17/5	na^4^
384	C	U	25/0	22/0	na
408	C	U	24/1	21/1	na
435	G	A/C	25/0	22/0	na
465	C	U	24/1	20/2	na
481	G	A	25/0	21/1	na
546	G	A/C	23/2	19/3	na
**Amino acid positions** 5					
70	R	non-R	7/18	5/17	5/21
91	L	M	3/22	5/17	6/19

1 Numbered relative to the start of the polyprotein.

2 Sequences obtained from [62].

3 Identified in [54], genotype 1b.

4 na, not available.

5 Identified in [55], [57].

Very similar results were obtained when we analyzed amino acid variation at core residues 70 and 91 (these codons include nucleotides 209 and 271, respectively). Having a residue other than arginine at position 70 or leucine at position 91 has been associated with HCC [55]–[57]. The majority of our HCC and cirrhotic sequences had the cancer signature at both positions 70 and 91 (Table 5). These results were corroborated by the external set of cirrhotic sequences. Therefore, the sequence patterns at all 11 nucleotide positions and both of the amino acid positions in core that have been previously associated with HCC were distributed almost identically among the HCC and cirrhotic sequences, with non-cancer sequence patterns predominating at 8 of the 11 nucleotide positions.

### Patient selection and sequencing to evaluate association of HCV genetic variation with rate of disease progression

The HALT-C trial evaluated the efficacy of long-term low-dose interferon α therapy on the rate of progression of liver disease in HCV patients who had previously failed interferon plus ribavirin therapy [68]. The study included a large observational control arm that did receive long-term interferon therapy, and hence provides a unique resource for studying HCV’s role in disease progression. Sixty patients from the observational arm of the HALT-C study who had been followed for four years were therefore identified for analysis; 30 were “slow progressors” and 30 were “rapid progressors”. All patients were infected with HCV subtype 1a, had failed prior interferon α plus ribavirin therapy, and had Ishak fibrosis scores at entry to HALT-C of 3 or 4. Patients co-infected with HBV or HIV were excluded. Patients were defined as “*Rapid Progressors*” if any of the standard HALT-C outcome criteria were met during the observation period: having a Child-Turcotte-Pugh (CTP) score ≥7 on two consecutive study visits, variceal hemorrhage, ascites, bacterial peritonitis, encephalopathy, advancement of the Ishak fibrosis score ≥2 points compared to the initial score, development of HCC, or dying from liver-related causes. “*Slow Progressors*” were defined as patients who did not meet any of these HALT-C outcomes during the observational period. The slow responders included 23 patients whose HCV titers never became undetectable during failed interferon-based antiviral therapy and 7 breakthrough or relapse patients. The rapid responders included 28 poor responders and 2 breakthrough/relapsers. The rapid and slow progressor groups were statistically indistinguishable at assignment to the control arm of HALT-C for an array of clinical parameters relevant to liver disease (Table 6).

**Table 6:** Baseline characteristics of patients from whom the rapid and slow progressor samples were derived.

	Slow progressors	Rapid progressors	P value^1^
Number of patients	30	30	–
Age (mean ± SD)	49.5±6.0	47.6±5.9	ns^2^
Female sex (% of patients)	26.7	43.3	ns
Duration of exposure to HCV (yr) (mean ± SD)	26.3±7.1	25.7±6.3	ns
Race or ethnic group (% of patients)^3^			ns
White	70.0	70.0	
Black	26.7	23.3	
Hispanic	3.3	6.7	
Body-mass index (BMI) (mean ± SD)^4^	28.6±5.2	31.8±7.6	ns
Diabetes (% of patients)	13.3	20.0	ns
Lifetime alcohol consumption (no. of drinks) (median)	8713	10516	ns^5^
Lifetime alcohol consumption (no. of drinks) (interquartile range)	2062–30286	1397–25913	
Baseline serum HCV RNA (log_10_ IU/ml) (mean ± SD)	6.5±0.4	6.5±0.5	ns
Serum alanine aminotransferase (ALT) (U/liter) (mean ± SD)	82.8±42.5	100.1±56.9	ns
Ratio of the patient’s alanine aminotransferase (ALT) level to the upper limit of normal (ULN)(mean ± SD)	1.7±1.1	1.8±0.9	ns
Total serum bilirubin (mg/dl) (mean ± SD)	0.7±0.3	0.8±0.5	ns
Serum albumin (g/dl) (mean ± SD)	4.0±0.3	3.9±0.4	ns
Prothrombin time (INR) (mean ± SD)	1.0±0.1	1.0±0.1	ns
Ishak fibrosis score^6^ (mean ± SD)	3.1±0.6	3.1±0.6	ns
Ishak inflammation score^7^ (mean ± SD)	7.7±2.0	7.5±1.8	ns
Esophageal varices (% of patients)	10.0	13.3	ns

1 T-test or chi-square test unless otherwise indicated.

2 Non-significant (p<0.05).

3 Race or ethnic group was self-reported.

4 BMI is the weight in kilograms divided by the square of the height in meters.

5 Wilcoxon rank-sum test.

6 The Ishak fibrosis score range is 0 (no fibrosis) to 6 (cirrhosis).

7 The Ishak inflammation score range is 0 (best) to 18 (worst).

The HCV open reading frame was sequenced for each patient from two time points separated by three years. Time point 1 (TP1) was nine months into the HALT-C observational period to allow HCV titers to rebound from failed interferon-based antiviral therapy that all patients received prior to randomization into the interventional or control arms of the study. The second time point (TP2) was at the end of the 45 month observational period. Consensus sequences for the HCV coding region were obtained from serum-derived RNA by direct sequencing of overlapping nested reverse transcriptase-PCR amplicons as previously described [26], [69], [70]. We were unable to sequence the full coding region from two rapid progressor and one slow progressor samples for time point 2, so these sequences were excluded from analyses involving time point 2.

### HCV positional differences associated with rate of disease progression

There were 15 positions in the TP1 and 13 positions in the TP2 sequences where the distributions of amino acids were significantly different between rapid and slow progressors, with seven of those positions overlapping between time points (Table 7). To help evaluate the likelihood that these may be spurious associations, we generated five sets of paired sequence groups where the 60 sequences were randomly assigned to one of two groups, with both groups containing 30 sequences. The number of positions that were significantly different between these pairs of randomized sequence sets ranged from 14 to 25, with a mean of 16.6. P-values ranged from 0.001 to 0.049, with a mean of 0.033. These values were very similar to the values seen when the rapid and slow progressor sequences were compared, suggesting that the differences in Table 7 are unlikely to reflect important biological variations associated with rate of disease progression.

**Table 7:** Positions where the distribution of amino acids differ between the rapid and slow progressor sequences.

Time point 1			Time point 2		
Position	Protein	P value	Position	Protein	P value
308	E1	0.038	308	E1	0.032
333	E1	0.040	391	E2	0.047
453	E2	0.031	464	E2	0.009
464	E2	0.028	853	NS2	0.034
490	E2	0.045	883	NS2	0.040
766	p7	0.038	1655	NS3	0.043
827	NS2	0.045	1723	NS4B	0.032
883	NS2	0.043	1747	NS4B	0.002
1723	NS4B	0.006	2047	NS5A	0.044
1746	NS4B	0.046	2361	NS5A	0.043
1747	NS4B	0.010	2369	NS5A	0.043
2181	NS5A	0.040	2414	NS5A	0.043
2185	NS5A	0.010	2482	NS5B	0.007
2361	NS5A	0.040			
2482	NS5B	0.012			

### HCV consensus sequence diversity differences are not associated with rate of disease progression

Pairwise genetic distances were calculated for the sequences in the rapid and slow groups for both TP1 and TP2 as we did for the cancer cohort. No significant differences were observed in the average pairwise distances between the rapid and slow progressors for either time point or between time points. Positions of variance relative to a population-wide reference were identified for rapid and slow progressors at both time points, and no significant differences were found at either time point between the two groups. This was true both when the entire polyprotein was evaluated as a single unit and when the viral genes were considered individually.

The paired sequences from TP1 and TP2 for each patient were compared and the numbers of mutations at the protein level were determined for each pair. There were no significant differences in the number of mutations during the three years between TP1 and TP2 between the rapid and slow progressors. This was true when the full polyprotein, each individual gene, or just the hypervariable regions 1 and 2 in E2 were compared.

### Selective pressures associated with disease progression

The rapid and slow progressor sequences were examined for codon selection differences using SLAC method as we did for the cancer cohort. Far more codons were under negative selection (∼1100 in both groups) than were under positive selection at both time points. The number of codons under positive selection was higher for slow progressors compared to rapid progressors at both time points (14 vs. 7 at TP1; 18 vs. 8 at TP2). Most of the codons under positive selection for the rapid progressors overlapped with those identified for the slow progressors (Table 8).

**Table 8:** Codons under positive selection in the rapid and slow progressor sequences.

Rapid progressors								
Time point 1					Time point 2			
Codon	Protein	Normalized dN-dS		P value	Codon	Protein	Normalized dN-Ds	P value
235	E1	3.751		0.002	235	E1	3.699	0.005
384	E2	5.284		0.007	397	E2	4.167	0.041
622	E2	2.491		0.017	522	E2	2.311	0.047
790	p7	2.054		0.033	570	E2	2.558	0.026
**1759**	**NS4B**	**2.112**		**0.026**	781	E2	3.021	0.018
2411	NS5B	1.854		0.046	**978**	**NS2**	**3.348**	**0.041**
2431	NS5B	2.287		0.022	2431	NS5B	2.798	0.021
					2963	NS5B	2.806	0.020
**Slow progressors**								
**Time point 1**					**Time point 2**			
**Codon**	**Protein**	**Normalized dN-dS**	**P value**		**Codon**	**Protein**	**Normalized dN-dS**	**P value**
235	E1	2.989	0.048		303	E1	3.363	0.044
384	E2	5.399	0.005		**384**	**E2**	**5.130**	**0.041**
395	E2	3.764	0.020		395	E2	4.359	0.037
396	E2	3.123	0.042		**445**	**E2**	**4.070**	**0.010**
**466**	**E2**	**3.400**	**0.005**		**466**	**E2**	**3.651**	**0.011**
522	E2	2.330	0.014		522	E2	3.288	0.008
622	E2	2.182	0.036		622	E2	2.718	0.035
781	p7	2.175	0.038		**641**	**E2**	**3.743**	**0.045**
2079	NS5A	1.984	0.045		790	p7	3.403	0.041
**2320**	**NS5A**	**3.491**	**0.023**		2079	NS5A	2.765	0.032
2411	NS5B	2.240	0.029		**2320**	**NS5A**	**4.150**	**0.025**
2537	NS5B	2.815	0.017		**2375**	**NS5A**	**2.493**	**0.040**
2600	NS5B	2.817	0.017		2411	NS5A	2.749	0.036
2729	NS5B	2.674	0.022		2431	NS5B	2.751	0.032
					2537	NS5B	4.094	0.009
					2600	NS5B	3.510	0.017
					2729	NS5B	3.282	0.022
					2963	NS5B	2.784	0.028

**Bold** indicates positions unique to HALT-C compared to random control alignments.

To help evaluate whether these differences in the number of codons under selection may be related to the rate of disease progression, we randomly selected six sets of 30 subtype 1a sequences from Genbank and examined their positive selection patterns. The number of sites under positive selection for the control sets ranged from 8 to 22 with an average 15.5. All but three of the codons for TP1 and seven of the codons for TP2 were under positive selection in one or more of the control sets (Table 8). Therefore, almost all of the sites under positive selection in the HALT-C dataset were not preferentially associated with the rate of disease progression. However, the slow progressor sequences were under greater positive selection pressure compared to the rapid progressor sequences.

### UU and UA dinucleotide frequency differences are not associated with rate of disease progression

The ratio of observed/predicted dinucleotide frequency was determined for every possible dinucleotide pair for each sample as before. As with the HCC cohort, all of the samples had reduced frequencies of UA and UU dinucleotides, but there were no significant differences in the observed/expected UU or UA ratios between the rapid and slow progressors, either within a time point or when the time points were combined. Only the AU dinucleotide had a statistically significant difference in the observed/predicted ratio between rapid (0.919) and slow (0.933) progressors (p<0.001). Although this is statistically significant, the magnitude of the change is very small and hence the difference unlikely to be biologically significant.

### Amino acid covariance patterns are not associated with rate of disease progression

Finally, we generated amino acid covariance networks for the HCV sequences from the rapid and slow progressors at both time points using the same methods used for the HCC cohort. As has been observed for other HCV sequence sets [27], [29], [31], [75], amino acid covariance networks were identified that involved residue positions from all 10 proteins and that had a hub-and-spoke topology. For both time points, network parameters including number of nodes, number of edges, mean number of neighbors, density and clustering coefficient were very similar between rapid and slow progressors (Table 9). About half of the covarying residue pairs and over 80% of the residue positions overlapped between rapid and slow progressor networks, indicating that the networks were very similar (data not shown). The networks generated at the two time points for the rapid progressors were almost indistinguishable, as were the two networks for the slow progressors. Therefore, covariance network analysis failed to identify differences between the rapid and slow progressor sequences.

**Table 9:** Basic amino acid covariance network parameters for the rapid and slow progressor sequences.

Network	Number of sequences	Number of nodes	Number of edges	Average number of neighbors	Network density
TP1-rapid	30	122	1226	20.1	0.17
TP1-slow	30	124	1163	18.7	0.15
TP2-rapid	28	122	1201	19.7	0.16
TP2-slow	29	123	1051	18.4	0.15

## Discussion

HCV is genetically very diverse, and viral genetic variation is a major contributor to virulence in many viral pathogens. However, evidence for or against HCV’s high genetic variation leading to differential virulence within a genotype is limited. Here, we examined HCV genetic variation in the full viral protein coding region to determine if genetic differences in HCV genotype 1 are associated with the development of HCC or the rate of disease progression. In sharp contrast to the strong associations we and others found between viral diversity and covariation patterns with response to interferon α-based therapy [25]–[27], [30], [31], very few HCV genetic associations were found with development of HCC or the rate of disease progression.

### HCV genetic associations with HCC

The HCC and cirrhotic control sequences were very similar, but we were able to identify two differences between them. First, there were 25 positions where the distribution of amino acids in the HCC and cirrhotic sequences were significantly different, which was more than the differences observed between control sequence sets in which these sequences were randomly re-sorted without regard for disease state. Four of these positions were within the p7 gene (Table 2). This clustering of differences within the very small p7 protein (63 residues) may imply a previously undefined role for this ion channel protein in the progression to HCC within a badly diseased liver. Three studies from Japan previously examined the entire HCV ORF for positions of variability associated with HCC [64], [65] [66]. These studies each identified up to nine positions in the core, E2, NS2, NS3 and NS5A genes where the amino acid distribution differed significantly between viruses from HCC patients and asymptomatic controls. The positions of skewed amino acid distributions we found were not the same as the sites found by the Japanese investigators. Together, these observations indicate that there may be some sites in the HCV genome where sequence differences are associated with HCC, but the inconsistency in the positions identified implies that it is unlikely such differences will be informative mechanistically or diagnostically. Second, we found eight positions under positive selection that were unique to either the HCC or cirrhotic control groups that were not under positive selection in randomly selected sets of genotype 1b sequences (Table 3). These positions may therefore be under evolutionary pressures associated with these advanced disease states.

Nucleotide sequence variations at eleven positions within the core gene have been previously associated with HCC [54], and amino acid variations at core positions 70 and 91 are associated with HCC in HCV 1b-infected patients, especially in Japan [55]–[57], [66]. However, sequences corresponding to the non-HCC signature strongly predominated at eight of these eleven nucleotide positions in both the cirrhotic control sequences and the HCC sequences. This observation was confirmed by evaluating an external set of cirrhotic patients [62] Table 5). The three exceptions were at nucleotides 78, 209 (within codon 70), and nucleotide 271 (in codon 91). Here, the HCC signature predominated in both the HCC and cirrhotic sequences. The previous studies that identified genetic associations in the HCV core gene with HCC used non-cirrhotic patients as controls [54], [58], but 80–90% of HCV-associated HCCs develop within a cirrhotic liver [82]. The equal prevalence of the cancer-associated genetic signatures in the HCC and cirrhotic control sequences indicates that these signatures are more likely to reflect an adaptation of HCV to a cirrhotic liver rather than direct associations with HCC.

We found no significant differences in the covariance networks between the HCC and cirrhotic controls. This result in is contrast to our previous covariance network analyses of HCV that identified strong signatures associated with early response to interferon-based treatment [27]. Furthermore, a different covariance algorithm has also identified associations with therapy outcome, gender and ethnicity of the patient [31]. The success of these methods when applied to data sets of similar size in finding associations with response to therapy but not with HCC implies that there are no strong HCV genome-wide genetic signatures specifically associated with HCC.

### HCV genetic associations with the rate of disease progression

The only substantial difference we detected between HCV sequences from the rapid and slow progressors was that the slow progressors were under greater positive selection than the rapid progressors (Table 8). The primary driver of positive selection in HCV is escape from adaptive immune responses [83], [84], and hence this result may reflect a waning of anti-HCV immunity in the deteriorating hepatic environment. It may also be related to reduced HCV antigen burden due to reduced HCV replication in the badly diseased liver tissue. The five other measures of genetic differences that we evaluated all failed to reveal significant differences between the rapid and slow progressor sequences at either of the two time points assessed. This lack of difference between the sequence sets, which includes the covariance networks, implies that any potential HCV genetic differences associated with the rate of disease progression must be smaller than the statistical power provided by sample sizes of 30 per arm. This in turn implies that HCV genetic differences are unlikely to be a dominant cause of differential disease progression in genotype 1a infected patients.

### Limitations and strengths and of this study

This study has four notable technical limitations. First, sample sizes were limited to 22–30 sequences per arm in the comparisons. This limited the statistical power in these analyses compared to larger studies that have focused on discrete regions of the HCV genome [54], [57], [62], [85]. Second, this is a cross-sectional retrospective study that cannot resolve whether the genetic patterns associated with HCC helped cause HCC or are viral adaptations to the neoplastic/cancerous environment. Third, the failure to identify HCV genetic sequence differences associated with rate of disease progression may have been partially affected by the fact that all HALT-C participants had failed prior interferon α plus ribavirin treatment. We and others have reported that HCV inter-patient genetic diversity is lowest among non-responders to interferon-based antiviral therapy [25], [26], [86]. This may limit the generality of the conclusions related to rate of disease progression. Finally, the HCV sequences were obtained from serum rather than from liver biopsies because liver samples were not available. The large majority of HCV in circulation is derived from hepatocytes, but differential genetic variability in core sequences from tumor tissue compared to core sequences from non-tumorous tissue has been demonstrated for some patients [87], [88].

This study has three strengths that permit substantial conclusions to be drawn despite the overall negative nature of the data. First, we employed two carefully selected sample sets derived from patients who had been matched with regard to HCV subtype, age, gender, and possible confounders of liver disease development in order to isolate effects on liver pathology associated with viral genetic variation within HCV genotype 1. Second, the study provided a comprehensive evaluation of HCV’s coding potential that was not blind to amino acid variations outside of a pre-determined target region. Third, we previously found strong genetic diversity differences between responders and non-responders to pegylated interferon α plus ribavirin therapy using these same methods on data sets of similar size that were derived from the Virahep-C study [25]–[27], [79]. For example, with samples sizes of 23–24 sequences per arm, we identified amino acid diversity differences in the core, NS3, and NS5A genes at p≤0.005 between early responders and non-responders to interferon-based treatment [26]. Therefore these methods can identify biologically significant viral genetic differences. This indicates that if viral genetic diversity differences existed between the HCC and control sequences or between the rapid and slow progressors, they must be substantially smaller than the viral genetic differences associated with response to interferon-based therapy.

### Concluding comments

The primary implications of this work stem from the contrast of the negative results from both of the pathology-related sequence data sets to the positive results from similar efforts focused on response to interferon-based therapy. This contrast implies that the differential rate of disease progression and HCC development among HCV patients is not strongly influenced by variability in HCV’s intrinsic ability to control the type 1 interferon response. It also implies that rapid disease progression and HCC do not have a large and/or consistent impact on HCV’s genetic patterns. Together, the lack of strong HCV genetic differences between HCC and cirrhotic patients and between rapid and slow disease progressors implies that host and/or environmental factors are the dominant causes of differential disease presentation in HCV patients.

## Supporting Information

Table S1 **List of sequence IDs, accession numbers and experimental groups for both patient cohorts.**
(XLSX)
